# Conceptualization of fibroblast growth factor receptor 1 targeting nanomedicines

**DOI:** 10.37349/etat.2025.1002353

**Published:** 2025-12-17

**Authors:** Yilin Ma, Mengqin Guo, Yang Liu, Zhengwei Huang

**Affiliations:** Peking University, China; ^1^Program of Pharmacy, International School, Jinan University, Guangzhou 510632, Guangdong, China; ^2^State Key Laboratory of Bioactive Molecules and Drugability Assessment, Guangdong Basic Research Center of Excellence for Natural Bioactive Molecules and Discovery of Innovative Drugs, College of Pharmacy, Jinan University, Guangzhou 511436, Guangdong, China; ^3^School of Pharmacy, Chongqing Medical and Pharmaceutical College, Chongqing 400016, China

**Keywords:** fibroblast growth factor receptor 1, anti-FGFR1 antibody, cancer targeting, nanomedicine

## Abstract

Fibroblast growth factor receptor 1 (FGFR1) is crucial in the progression of various cancers, participating in the processes of cell proliferation, survival, and differentiation. FGFR1 plays a role in the resistance to immune checkpoint inhibitors (ICIs) such as pembrolizumab and nivolumab. Therefore, using monoclonal antibodies and tyrosine kinase inhibitors to target FGFR1 and enhancing ICIs by modifying the tumor microenvironment and combating immune suppression represents a potential therapeutic strategy. Based on the FGFR1-related research and the active targeting strategy, we believe that modifying the surface of nanomedicines with anti-FGFR1 antibodies (such as OM-RCA-01) is an effective targeted treatment method for tumors with high expression of FGFR1. Although there have been relevant studies confirming the feasibility of this approach, there are challenges in clinical application, especially in terms of maintaining uniform quality during large-scale production. Therefore, we suggest conducting further optimization studies in the future to accelerate the clinical application of such drug delivery systems and provide more efficient and cost-effective options for tumor treatment.

## Main text

**Figure 1:**
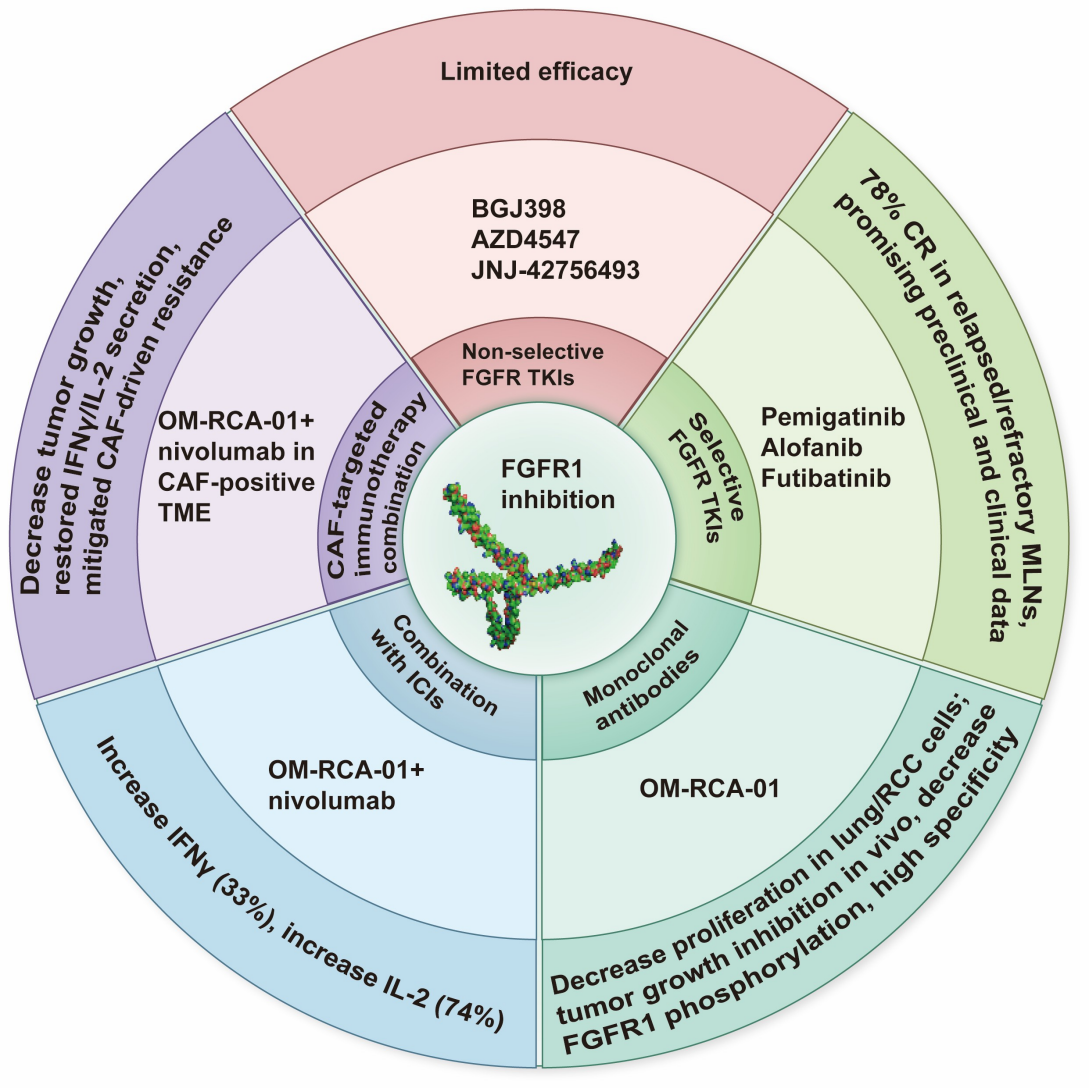
**Potential therapeutic strategy of FGFR1 inhibition.** FGFR1: fibroblast growth factor receptor 1; ICIs: immune checkpoint inhibitors; CR: complete response; MLNs: myeloid/lymphoid neoplasms; CAF: cancer-associated fibroblast; TME: tumor microenvironment; TKIs: tyrosine kinase inhibitors; RCC: renal cell carcinoma.

In a newly published Perspective article by Ilya Tsimafeyeu [1], the author summarized that fibroblast growth factor receptor 1 (FGFR1) is crucial in the progression of various cancers, participating in the processes of cell proliferation, survival, and differentiation. Recent research findings indicate that FGFR1 is also related to the mechanism of immune evasion. Especially, it plays a role in the resistance to immune checkpoint inhibitors (ICIs) such as pembrolizumab and nivolumab [2]. Therefore, targeting FGFR1 with monoclonal antibodies and tyrosine kinase inhibitors has become a potential therapeutic strategy to enhance the efficacy of ICIs by altering the tumor microenvironment and combating immune suppression. Of note, OM-RCA-01, a monoclonal antibody for FGFR1, has been proposed as a targeting agent. Preclinical studies have shown that the combination of novel FGFR1 inhibitors, such as the monoclonal antibody OM-RCA-01, with ICIs can significantly enhance antitumor activity, boost T-cell responses, and cytokine production [3]. This article discussed the role of FGFR1 in cancer biology, its impact on resistance to immunotherapy, and the therapeutic potential of targeting FGFR1 to enhance the efficacy of ICIs, providing a research foundation for the mechanism of FGFR1 in tumor immune escape and clinical reference significance for optimizing the combined treatment scheme of FGFR1 targeting and immunotherapy (Figure 1).

### Design conceptualization

Drawing inspiration from such a perspective article, on top of our research experience, we would like to reanalyze the connotation between the acquired knowledge and nanomedicine design and development. We expect that with our recommendations, the meaning and significance of the original paper could be further enhanced.

**Figure 2:**
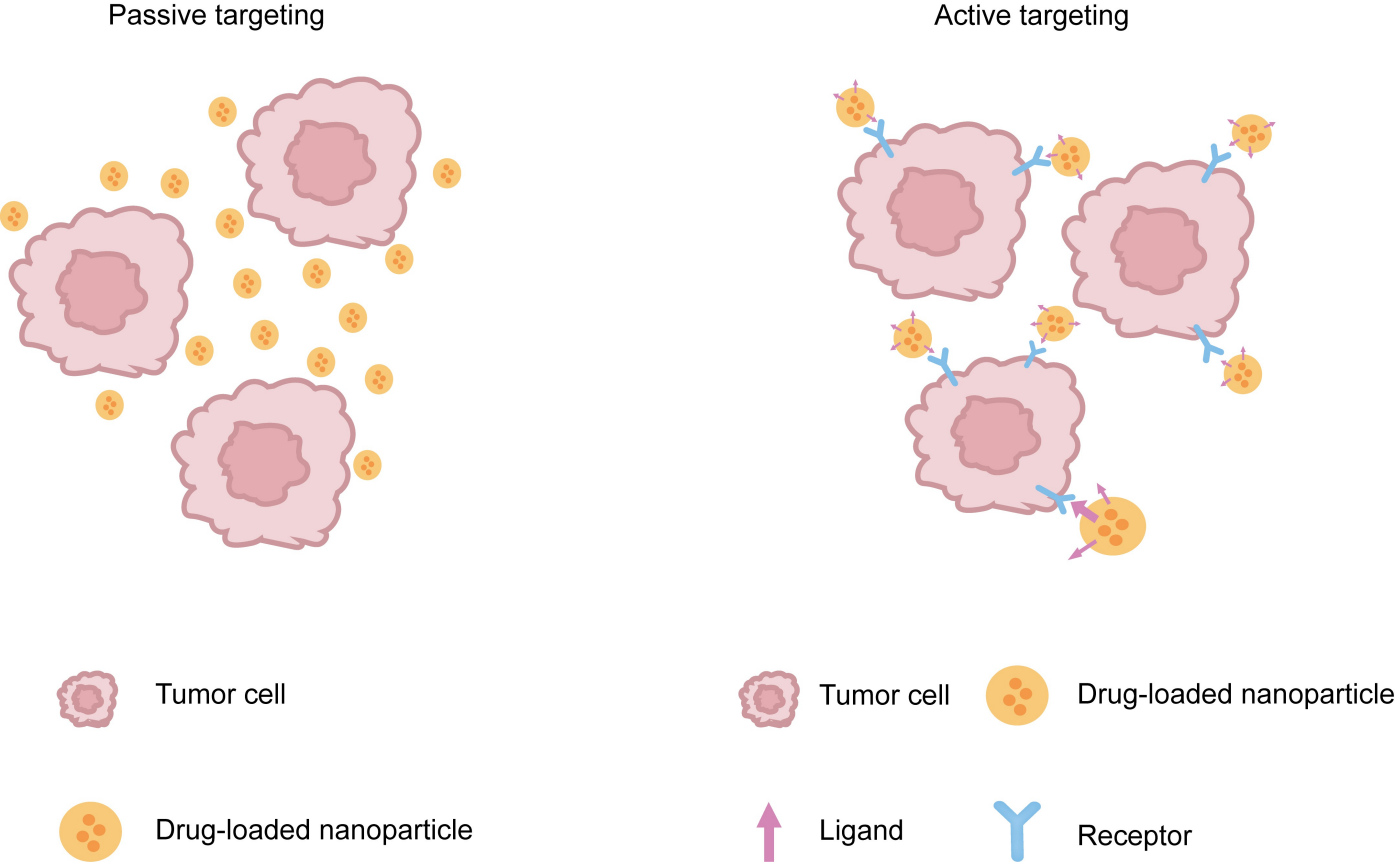
Sketch illustrating active and passive targeting of anti-FGFR1 [17].

Commonly reported nanomedicines include nanoliposomes, solid lipid nanoparticles, nanocapsules, nanospheres, and polymer micelles [4]. Upon the targeting mechanisms of nanomedicines, there are mainly two approaches: passive targeting and active targeting. Passive targeting refers to a drug delivery strategy that leverages the physicochemical properties of nanomedicines—including particle size, surface characteristics, and blood half-life—to achieve targeted distribution through natural physiological mechanisms within the biological system. For example, in a study by Li et al. [5], the deposition of the nanocarriers with a molecular weight of approximately 80 kDa at the tumor site was achieved by utilizing the selectivity increased in blood flow when blood pressure rose due to the lack of a smooth muscle layer in tumor microvessels, the wide endothelial space of blood vessels and the incomplete basement membrane, as well as the defect of lymphatic drainage. It was achieved by combining the inherent characteristics of the nanocarrier, which has a molecular weight exceeding the renal excretion threshold (40 kDa) to avoid rapid clearance, and was adapted to the size of the tumor vascular leaky structure to achieve selective penetration [6]. Active targeting is a method that enhances the targeting property of nanomedicines by modifying specific targeting ligands (such as antibodies, peptide chains, receptor-specific molecules, etc.) on the surface of the nanoparticles. It utilizes natural phenomena in the bodyʼs physiological environment to achieve targeted drug delivery (Figure 2).

As Jariwala et al. [7] stated: Surface functionalization is one of the approaches for modification, which is defined as the introduction of chemical moieties (functional groups or other ligands) on the surface of any material to imbibe the desired characteristics, as well as the differences between functionalized biological materials and non-functionalized biological materials: The surface of non-functionalized materials is heterogeneous and has poor performance, which imposes many limitations in biomedical applications. The biocompatibility of functionalized materials is significantly improved compared to non-functionalized materials. Active targeting and increased loading efficiency are achieved, providing a platform for efficient diagnosis and treatment. As comprehensively reviewed by Mitchell et al. [8], the anti-adhesion strategy: The internal polymer stabilizes the structure and controls the drug release. The outer layer has a natural cell membrane, enabling evasion of immune detection, prolonging circulation time, and ensuring the nanoparticles reach the lesion site, achieving efficient delivery. Biocide-attachment release strategy: The chemotherapy drugs (anti-cancer “bactericidal” agents) are encapsulated within LPHNPs. They remain stable during circulation and are precisely released upon specific internal stimuli in the tumor microenvironment (such as overexpressed enzymes). This not only kills cancer cells but also reduces systemic toxicity. Contact-active biomaterial strategy: The contact-active ligand ensures specific binding to cells, enabling specific recognition of the receptors overexpressed on the cancer cell membrane. This direct contact not only enhances cell uptake but also triggers specific biological signal transduction, thereby achieving better therapeutic effects.

For example, in a series of studies [9], anti-CD19 Fab' antibody fragments that recognize B-cell antigen CD19 were used as targeting ligands modified on the surface of PEGylated liposomes. In this way, the nanoparticles were preferentially taken up by tumor cells through the specific interaction between the ligands and the antigens [10]. Due to the numerous limitations of passive targeting, such as the lack of specific recognition of tumor cells, the targeting efficiency of passive targeting is usually limited, while that of active targeting is relatively higher. It is better to design targeting nanomedicines using the active targeting method [11].

In active targeting, the targeting ligand is the key to providing targeting moieties. Among them, specific antibodies are an excellent targeted ligand [12]. OM-RCA-01, as an anti-FGFR1 antibody, has the effect of specifically targeting FGFR1 that is overexpressed on the cell surface. Therefore, it can be used as an active targeting ligand. Functionalized adjustable drug release can be achieved, for instance, through pH-responsive or enzyme-sensitive linkers for controlled release, thereby enhancing efficacy and reducing side effects. Given that non-small cell lung cancer (NSCLC), breast cancer, glioma, prostate cancer, head and neck tumors, renal cell carcinoma, and other tumor cells have high surface expression of FGFR1, on the nanomedicine's surface, modifying anti-FGFR1 antibodies can endow excellent tumor-targeting performance [3]. It is expected to achieve improved targeted therapeutic efficacy for tumor management by this strategy. We draw on the successful research about the various biological effects that can be provided by nanomedicines decorated with anti-FGFR antibodies by Nguyen et al. [13]. (i) Enhancing the endocytosis of cells: The binding of antibodies to FGFR1 can trigger receptor-mediated endocytosis, and nanoparticles can quickly enter the cells. For instance, in the EGFR-targeting scenario, the nanoparticles conjugated with cetuximab showed a significantly higher internalization rate in cells with overexpressed EGFR compared to non-targeted particles. Therefore, we expect that the anti-FGFR1 nanoparticles will exhibit a similar enhancement of uptake in FGFR1-positive tumor cells. (ii) Increasing tumor accumulation and retention: Compared with H1299 cells with low-level EGFR expression, the therapeutic effect of cetuximab-coated gold nanoparticles was more significant in A549 cells with overexpressed EGFR, indicating that cetuximab achieved selective targeting. Specific binding not only promotes cell uptake but also cooperates with active targeting through the EPR effect to enhance the enrichment and retention of nanoparticles at the tumor site, thereby increasing the local drug concentration. (iii) Improving therapeutic index and reducing systemic toxicity: By delivering therapeutic drugs specifically to tumor cells, targeted nanomedicines can enhance the killing of cancer cells while minimizing off-target effects and toxicity to normal tissues. Furthermore, antibody modification may regulate the immune response, for example, by targeting FGFR1 to influence the balance of pro-inflammatory/anti-inflammatory cytokines in the tumor microenvironment, but further research is needed.

**Figure 3:**
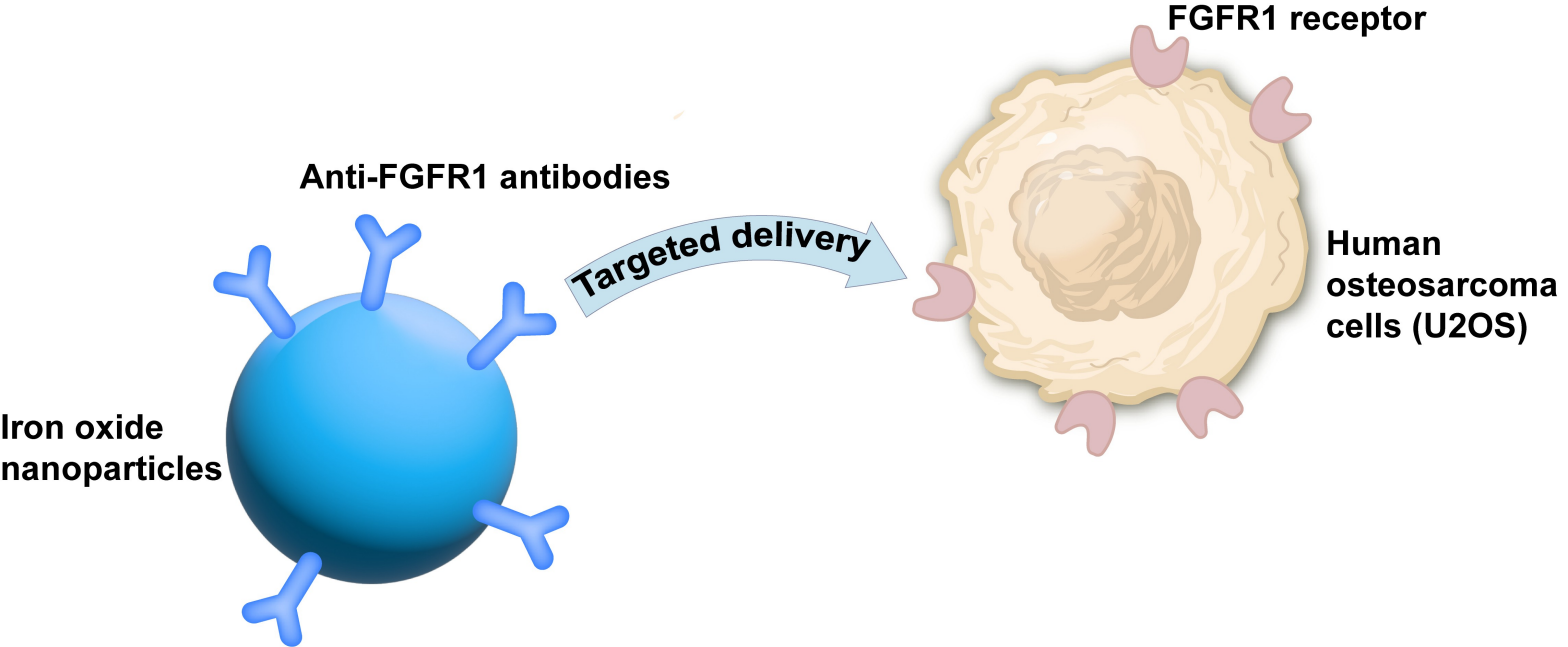
**Study using anti-FGFR1 antibodies to modify iron oxide nanoparticles to achieve the treatment of human osteosarcoma cells (U2OS) with FGFR1 overexpression tumors with a selective killing effect [14].** FGFR1: fibroblast growth factor receptor 1.

After a literature survey, we have found that there are currently relevant studies. For instance, researchers used anti-FGFR1 antibodies to modify iron oxide nanoparticles to achieve the treatment of human osteosarcoma cells (U2OS) with FGFR1 overexpression tumors with a selective killing effect (Figure 3) [14].

From the perspective of investigational new drug development, it should be noted that such a modification is different from preparing an antibody-drug conjugate (ADC). ADC refers to a class of targeted therapeutic systems that combine monoclonal antibodies with cytotoxic drugs through a linker [15], essentially belonging to another type of new chemical entity (NCE). Therefore, in the FDAʼs 505b (1) or China NMPAʼs new drug application process, the safety and efficacy of ADC needs to be re-studied independently of the drug itself [16]. Our proposed idea is to incorporate anti-FGFR1 antibodies onto the surface of nanoparticles through physical or chemical methods, thereby creating a new type of drug delivery system without the need to develop a new NCE. The physical methods include adsorption or encapsulation (such as based on hydrophobic interaction); the chemical methods include covalent coupling (such as using EDC/NHS to link the amine groups of the antibody to the carboxyl groups of the nanoparticles). The physical methods are simple but have poor stability, while the chemical methods are stable but require control of the coupling efficiency. For drugs that have already been approved by the FDA, developing an anti-FGFR1 antibody-modified nano-delivery system is essentially modified formulation research (equivalent to the FDAʼs 505b (2) approach or the modified new drug application of China NMPA). The previous research data on the safety and efficacy of the drug itself are still applicable, and thus the cost is relatively low. Hence, for the sake of new drug application, we believe that the design concept of the anti-tumor nanomedicine proposed in this article has certain reference value.

Of course, constructing a nanodelivery system modified with anti-FGFR1 antibodies also involves certain technical difficulties. In particular, how to maintain the quality homogeneity and reproducibility under large-scale production is worthy of in-depth discussion. We suggest focusing on the following issues: Improving antibody modification methods to enhance the stability of antibody-nanoparticle conjugation during the synthesis process; formulating a quality control plan to accelerate the promotion of the clinical application of related drug delivery systems.

## Conclusions

Based on the FGFR1-related research and the active targeting strategy, we believe that modifying the surface of nanomedicines with anti-FGFR1 antibodies (such as OM-RCA-01) is an effective targeted treatment method for NSCLC, breast cancer, glioma, prostate cancer, head and neck tumors, renal cell carcinoma, and other tumors with high expression of FGFR1. Compared with passive targeting, the active targeting approach can significantly improve the targeting efficiency. Compared with ADC, as a new drug delivery system, it can utilize the safety and efficacy data of existing NCEs in the new drug application process, reducing the research and development costs, and better conforming to the FDA 505b (2) or the improved new drug application path of Chinaʼs NMPA.

We analyze the possible limitations and strengths of Ilya Tsimafeyeu’s [1] work; its strength is that it clarifies the mechanism by which FGFR1 drives immune resistance, proposing the synergistic treatment of “FGFR1 targeting + immunotherapy”; it also provides direct anti-tumor data of OM-RCA-01 in vitro and in vivo. However, a limitation is the absence of a treatment arm using OM-RCA-01 alone.

Although there have been relevant studies confirming the feasibility of this approach, such as researchers using anti-FGFR1 antibodies to modify iron oxide nanoparticles to achieve the treatment of U2OS with FGFR1 overexpression tumors with a selective killing effect, there are challenges in clinical application, especially in terms of maintaining uniform quality during manufacturing. Therefore, we suggest conducting further optimization studies in the future to accelerate the clinical application of such drug delivery systems and provide more efficient and cost-effective options for tumor treatment.

Notwithstanding the above comments, we highly appreciate the insightful contribution by Ilya Tsimafeyeu.

## Abbreviations

ADC: antibody-drug conjugate
FGFR1: fibroblast growth factor receptor 1
ICIs: immune checkpoint inhibitors
NCE: new chemical entity
NSCLC: non-small cell lung cancer

## Declarations

### Copyright

© The Author(s) 2025.
